# Elevation of neutrophil carcinoembryonic antigen‐related cell adhesion molecule 1 associated with multiple inflammatory mediators was related to different clinical stages in ischemic stroke patients

**DOI:** 10.1002/jcla.24526

**Published:** 2022-06-03

**Authors:** Yi Zhang, Yijie Wang, Wei Wu, Ping Liu, Shanshan Sun, Meng Hong, Yuan Yuan, Qi Xia, Zhi Chen

**Affiliations:** ^1^ Department of Laboratory Medicine The First Affiliated Hospital, Zhejiang University School of Medicine Hangzhou China; ^2^ Key Laboratory of Clinical In Vitro Diagnostic Techniques of Zhejiang Province Hangzhou China; ^3^ State Key Laboratory for Diagnosis and Treatment of Infectious Diseases, National Clinical Research Center for Infectious Diseases, Collaborative Innovation Center for Diagnosis and Treatment of Infectious Diseases The First Affiliated Hospital, Zhejiang University School of Medicine Hangzhou China; ^4^ Department of Neurology The First Affiliated Hospital, Zhejiang University School of Medicine Hangzhou China

**Keywords:** apoptosis, CEACAM1, interleukin, ischemic stroke, neutrophils

## Abstract

**Background:**

We aimed to analyze the level of carcinoembryonic antigen‐related cell adhesion molecule 1 (CEACAM1) in neutrophils of ischemic stroke (IS) patients at different stages, together with its roles in neutrophils.

**Patients and methods:**

Sixty‐seven patients were classified into acute phase group (*n* = 19), subacute phase group (*n* = 28), and stable phase group (*n* = 20), and 20 healthy individuals who had received physical examination at the same time period as healthy control. We then analyzed the expression level of CEACAM1 and cell viability in CEACAM1 positive and CEACAM1 negative neutrophils by flow cytometry and the content of plasma CEACAM1, neutrophil gelatinase‐associated lipocalin (NGAL), matrix metalloproteinases‐9 (MMP‐9) was measured using enzyme‐linked immunosorbent assay (ELISA), while that of interleukin‐10 (IL‐10) and tumor necrosis factor (TNF) was determined using a Human Enhanced Sensitivity Flex set.

**Results:**

Compared with healthy control, the percentage of CEACAM1 positive neutrophils in IS patients showed a significant increase, and a significant increase was also noticed in the content of plasma CEACAM1 at the subacute stage. Reduction in cell viability was observed in CEACAM1 positive neutrophils compared with CEACAM1 negative counterparts. There was a positive correlation between CEACAM1 expression rate in neutrophils and plasma CEACAM1 and IL‐10 content in the subacute group. Compared with acute group and healthy control group, there was an instinct increase in the level of plasma MMP‐9 and NGAL in subacute group.

**Conclusions:**

Our data showed that there was a rapid increase of CEACAM1 in neutrophils at the acute stage of IS. We speculated that CEACAM1 may serve as an inhibitory regulator involving in the progression of IS.

## Graphical abstract

Ischemic stroke is associated with several factors. This study aims to analyze the level of carcinoembryonic antigen‐related cell adhesion molecule 1 (CEACAM1) in neutrophils at different stages of ischemic stroke (IS), together with the roles of CEACAM1 on neutrophils. Our data showed that there was rapid elevation of CEACAM1 in the neutrophils at the acute stage of IS. Reduction of cell viability was observed in CEACAM1 positive neutrophils compared to CEACAM1 negative counterparts. Moreover there was a positive correlation between CEACAM1 expression rate in the neutrophils and plasma CEACAM1 and IL‐10 content in the subacute group. We speculated that CEACAM1 may serve as an inhibitory regulator involving in the progression of IS.

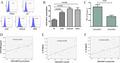

## 1 INTRODUCTION

Stroke, with an estimated global lifetime risk of 24.9%, presents an increasing trend worldwide.^1^ As the third major cause for disability‐adjusted life‐years,^2^ it shows a high mortality and morbidity.^3^ Ischemic stroke (IS), accounting for 80%–85% of the stroke population, is mainly associated with several factors, especially the thromboembolic occlusion in the cerebral artery. Upon onset of IS, many patients present hypoxic damages and excitotoxicity. Subsequently, a majority would show massive accumulation and infiltration of inflammatory cells during ischemic reperfusion that may trigger further brain injuries.^4^, ^5^ Meanwhile, human body would persistently release the anti‐inflammatory factors to defend the excessive inflammation to maintain the homeostasis. Thereafter, the immune system showed gradual failure, which finally entered into systemic immunosuppression,^6^, ^7^, ^8^ possibly leading to stroke‐associated infection (SAI).^9^, ^10^ Susceptibility to infection may severely hamper the prognosis and is mainly responsible for the increased mortality among IS patients.^6^, ^11^ On this basis, patients with cerebral ischemia may present immunological function disorders, especially the functional disorder of the innate immunocytes associated with the early‐stage blood–brain barrier (BBB) injury and late‐stage SAI.^12^ Nevertheless, little is known about the exact mechanisms in this process.

Neutrophils are the major effector cells of the innate immunity system. Upon IS onset, the neutrophils were the first type of cells adhered to the cerebral endothelium and infiltrated to the ischemic brain in the presence of cell adhesion molecules and chemotactic factors.^13^ Besides, it would lead to BBB and nervous system injuries through releasing the inflammatory mediators including proinflammatory cytokines, matrix metalloproteinases, and reactive oxygen species.^14^ The circulating leukocytes would infiltrate into the lesion sites after onset of IS in the presence of proinflammatory factors and chemotactic factors, which then further led to BBB injury through releasing proinflammatory cytokines, reactive oxygen species and matrix metalloproteinases (MMPs), together with formation of the vasogenic edema.^15^, ^16^, ^17^ In the previous study, MMP‐9 was considered to play an important role in the BBB injury after IS, while the MMP‐9 inhibitor provided protection against potential alternations in BBB permeability. Furthermore, increased serum IL‐10 and MMP‐9 was observed in patients with IS, among which IL‐10 was found to play an important anti‐inflammatory role in responding to brain injury.^18^, ^19^, ^20^, ^21^ Previous study showed that carcinoembryonic antigen‐related cell adhesion molecule 1 (CEACAM1) played a crucial role in the immune regulation.^22^, ^23^ As a member of the immunoglobulin superfamily, CEACAM1 is known as an inhibitory immune co‐receptor suppressing the signal transmission via two immunreceptor tyrosine‐based inhibition motifs.^23^, ^24^ Moreover, it is closely related to the proliferation and apoptosis of neutrophils and lymphocytes,^25^, ^26^, ^27^ together with the secretion of cytokines.^28^, ^29^ Our previous study indicated that there was significant increase in T‐cell immunoglobulin and mucin‐domain containing‐3 (TIM‐3) in CD8^+^ T cell in the IS patients,^30^ and CEACAM1 was also co‐localized with TIM‐3.^31^ Neutrophil gelatinase‐associated lipocalin (NGAL) is reported to involve in the innate immunity, cellular differentiation and proliferation, and iron homeostasis.^32^ Besides, it may form a complex with MMP‐9.^33^ Thus, CEACAM1 could directly or indirectly involve in the innate and regulatory immunity. In this study, we aimed to analyze the counts of neutrophils and the level of the serum CEACAM1, MMP‐9, and NGAL in the IS patients and analyze their possible roles in the IS process.

## 2 METHODS

### 2.1 Study subjects

Sixty‐seven IS patients admitted in the First Affiliated Hospital, College of Medicine, Zhejiang University were randomly recruited in this study. The diagnosis of IS was conducted using criteria issued by the World Health Organization Multinational Monitoring of Trends and Determinants in Cardiovascular Disease (WHOMONICA).^34^ IS was verified by magnetic resonance imaging (MRI) or computed tomography (CT). All the patients were classified into the following groups according to the time of post‐stroke: (i) acute phase group (within post‐IS 48 h); (ii) subacute phase group (post‐IS, 48 h to 10 days), and (iii) stable phase group (post‐IS, 10–30 days).^30^ Clinical data including sex, race, age, alcohol use status, smoking status, other disease status, and the National Institutes of Health Stroke Scale (NIHSS) score were recorded for the IS subjects.^35^ Infection was defined as presence of clinical symptoms including pyuria for urinary tract infection, fever, productive cough, positive bacterial culture, and radiographic evidence of consolidation for pneumonia. The exclusion criteria were as follows: those with a history of cerebral abscess, cerebral hemorrhage, transient ischemic attack, hematopathy, autoimmune diseases, cancer and pregnancy, trauma, or surgery within the last 3 months, severe infection in the recent 6 months, together with a history of human immunodeficiency virus (HIV) infection. Patients with hepatic and renal dysfunction were excluded from this study. In addition, we included 20 healthy individuals who had received physical examination in the First Affiliated Hospital of Zhejiang University at the same time period as healthy control. Table 1 summarized the demographic and clinical characteristics of subjects. Written informed consent was obtained from each subject. The procedures of this study were approved by the Ethical Committee of the First Affiliated Hospital, College of Medicine, Zhejiang University, and this study was performed in accordance with the Declaration of Helsinki.

**TABLE 1:** Demographics and baseline characteristics of patients with IS of different clinical stages

Variables	Control group (*n* = 20)	Acute phase group (*n* = 19)	Subacute phase group (*n* = 28)	Stable phase group (*n* = 20)	*p*
Age (years)	55.75 ± 11.36	58.26 ± 16.10	68.11 ± 11.59	66.67 ± 11.74	n.s.
Male %	55.00%	52.63%	60.71%	55.00%	n.s.
Race, Han, % (*n*)	100	100	100	100	n.s.
NIHSS score	NA	6.11 ± 4.03	3.47 ± 2.05	1.17 ± 1.17	*,#,∆
Hypertension, % (*n*)	0.00	63.16	67.86	65.00	n.s.
Diabetes mellitus, % (*n*)	0.00	36.85	32.14	35.00	n.s.
Hypercholesterolemia, % (*n*)	0.00	52.63	53.58	40.00	n.s.
Smoking status (% current smokers)	30.00	42.11	39.29	40.00	n.s.

*Acute phase group vs. sub‐acute phase group, *p* = 0.021. # Acute phase group vs. stable phase Group, *p* < 0.001. ∆ Subacute phase group vs. stable phase Group, *p* < 0.001. Chi‐square test was performed in all comparisons except for those on age and NIHSS score. Chi‐square test was performed in all comparisons except for those on age and NIHSS score. NIHSS score, National Institutes of Health Stroke Scale.

### 2.2 Flow cytometry

**FIGURE 1:**
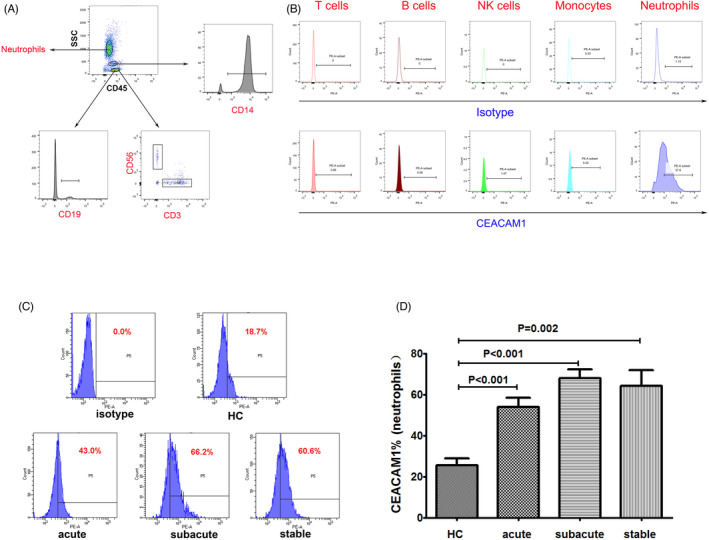
Analysis of CEACAM1 on neutrophils. (A) The T cells (CD3^+^), B cell (CD19^+^), NK cells (CD3^−^CD56^+^), monocyte (CD14^+^), and neutrophils were selected from the CD45 positive cells. (B) Expression of CEACAM1 in the IS patients. (C) Flow cytometry gating strategy for the determination of CEACAM1 on neutrophils. (D) Comparison of percentage of CEACAM1 positive cells in peripheral neutrophils in the IS patients and normal individuals. A post hoc test was utilized for the analysis. CEACAM1, carcinoembryonic antigen‐related cell adhesion molecule 1. NK cells, natural killer cells

The gating strategy of different immune cell populations is shown in Figure 1A and Figure S1. Briefly, the frequency of lymphocytes, neutrophils, and monocytes in the leukocyte population was determined based on their CD45 (APC BD Bioscience)‐SSC profile. T lymphocytes were CD45^+^CD3^+^ (BV421 BD Bioscience), and B lymphocytes were CD45^+^CD19^+^ (FITC Biolegend). The NK cells were CD45^+^CD3^−^CD56^+^ (APC, BD Bioscience). Monocytes were CD45^+^CD14^+^ (FITC BD Bioscience). The cells were analyzed using CEACAM‐1 (PE R&D). Isotype‐matched immunoglobulins served as control. About 30 min later, TQ‐PREP (Beckman Coulter) was used to lyse the heparinized blood. Flow cytometry (FACS CANTO II, Becton Dicknson,) was used for the analysis of blood samples in this study.

### 2.3 Cell viability

The heparinized blood was lysed using TQ‐PREP before the neutrophils were labelled with Annexin‐V‐FITC (BD Bioscience) and CEACAM1 (PE R&D). Flow cytometry was utilized to detect the CEACAM1 positive cells and the cells negative for apoptosis (FACS CANTO II, Becton Dicknson).

### 2.4 Measurement of plasma CEACAM‐1, NGAL and MMP‐9

The concentrations of plasma or poor‐plate plasma (targeted MMP‐9) were determined using a commercial ELISA Kits. CEACAM‐1 (DY2244) and MMP‐9 (DMP900) were purchased from R&D, while the NGAL (443407) was purchased from Biolegend. The absorbance was determined at 450 nm using an ELISA reader (iMark Microplate Reader, Bio‐Rad). The limitation of detection for CEACAM‐1, MMP‐9, and NGAL was 93.8 pg/ml, 0.156 ng/ml, and 16.4 pg/ml, respectively.

### 2.5 Intracellular MMP‐9 assays

The neutrophils were labelled with CEACAM1‐PE (R&D) at room temperature for 30 min and then were resuspended with 1 ml PBS. LPS (100 μg/ml, Sigma‐Aldrich) and Brefeldin A (BFA, Biolegend) were used to inhibit cytokine trafficking. About 2 h after incubation, the cells were fixed and permeabilized (Biolegend), followed by staining with MMP‐9‐FITC (R&D), at room temperature for 30 min. Finally, flow cytometry was conducted to analyze the intracellular MMP‐9.

### 2.6 Plasma cytokine assays

The concentrations of plasma TNF and IL‐10 were determined using a Human Enhanced Sensitivity Flex set (BD Bioscience). The lower limits of the test for the detection of the cytokines were 67.3 fg/ml for TNF and 13.7 fg/ml for IL‐10.

### 2.7 Statistical Analysis

We used the SPSS 22.0 software for the data analysis. The data were presented as the mean ± standard deviation. The normal distribution of data was determined based on the descriptive statistics. A one‐way analysis of variance (ANOVA) was utilized for the comparison of data that were normally distributed. Inter‐group difference was determined using the post hoc test. Kruskal–Wallis was utilized for the analysis of data that were not normally distributed. The difference in independent data between two groups was analyzed by the Mann–Whitney nonparametric test. The Pearson correlation analysis was performed to assess associations among plasma TNF, IL‐10, CEACAM1 content, and CEACAM1 positive neutrophils in the IS group. Statistical difference was considered in the presence of *p* < 0.05.

## 3 RESULTS

### 3.1 Frequency of CEACAM1‐positive cells on the surface of neutrophils in the peripheral blood

Flow cytometry validated the expression of CEACAM1 in the white blood cells of peripheral blood in IS patients. CEACAM1 was mainly expressed on the surface of neutrophils. In contrast, CD3^+^ T cells, natural killer (NK) cells, monocytes, and B cells expressed low levels of CEACAM1 (Figure 1A and B). As shown in Figure 1C and D, the percentage of CEACAM1‐positive cells on the surface of neutrophils in the peripheral blood in IS groups showed a significant increase compared with that of the control group (*p* < 0.01). There were no statistical differences in the percentage of CEACAM1‐positive cells on the surface of neutrophils among the acute group, subacute group, and stable group (*p* > 0.05).

### 3.2 Apoptotic phenotype of CEACAM1‐positive and negative neutrophils

**FIGURE 2:**
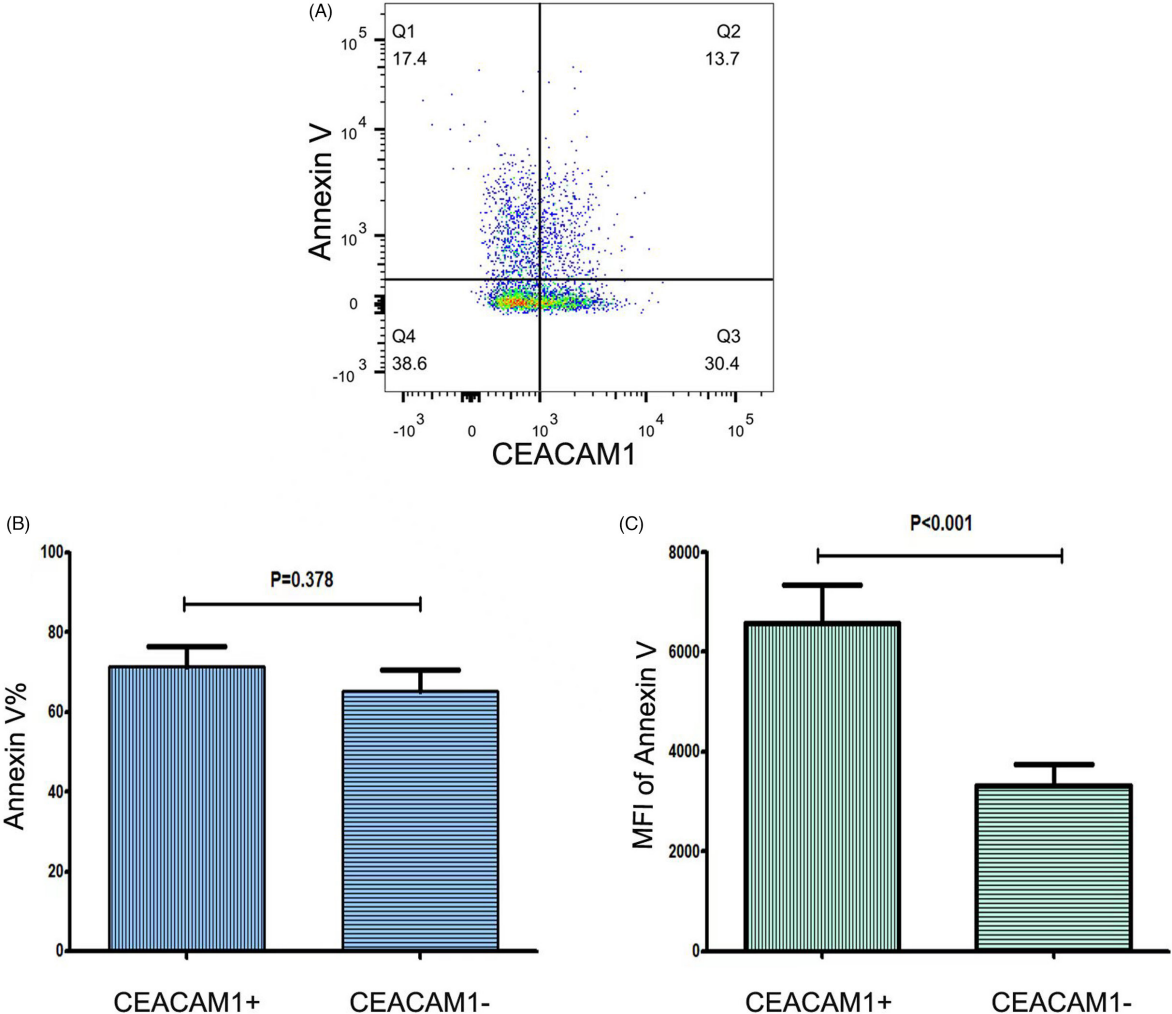
Expression of Annexin‐V in the CEACAM1 positive and negative cells in patients from the subacute group(*n* = 10). (A) Gating strategy of Annexin‐V. (B) Percentage of Annexin‐positive cells. There was no statistical difference between the CEACAM1 positive and negative cells. (C) MFI of Annexin‐V. There was a significant difference between the CEACAM1 positive and negative cells. Statistical analysis was performed by the Mann–Whitney test. CEACAM1, carcinoembryonic antigen‐related cell adhesion molecule 1

To confirm the correlation between CEACAM1 expression and the apoptosis of neutrophils, flow cytometry was utilized to determine the positive rate of Annexin‐V in the CEACAM1 positive and negative neutrophils. As shown in Figure 2A and B, in patients of subacute stage, Annexin‐V was expressed in both CEACAM1 positive and negative neutrophils. We calculated the percentage of Annexin‐V‐positive cells and mean fluorescence intensity (MFI) of Annexin‐V separately of CEACAM1‐positive and negative cells. The result showed that there was higher Annexin‐V MFI in CEACAM1‐positive cells (*p* < 0.001), which suggested reduction in cell viability of CEACAM1‐positive cells compared to negative counterparts.

### 3.3 Determination of plasma soluble CEACAM1

**FIGURE 3:**
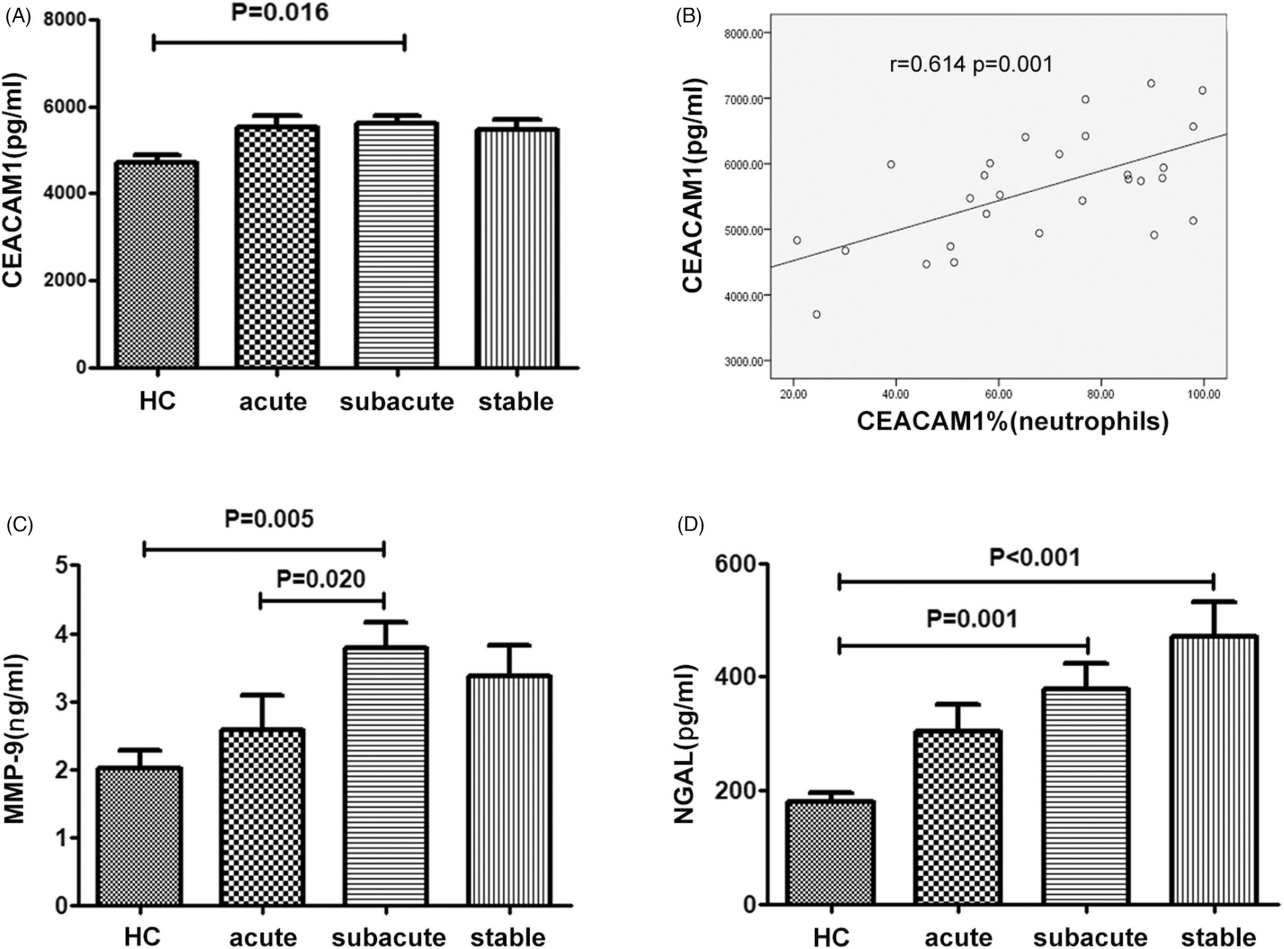
Content of CEACAM1, NGAL and MMP‐9 in the plasma of patients with IS. (A) Comparison of plasma CEACAM1 concentration in the IS patients and healthy control. (B) Correlation analysis between plasma CEACAM1 content and percentage of CEACAM1 positive neutrophils in the subacute group. (C, D) Comparison of plasma MMP‐9 and NGAL in the IS group and the healthy control group. A post hoc test or Kruskal–Wallis was utilized for the analysis. CEACAM1, carcinoembryonic antigen‐related cell adhesion molecule 1. NGAL, neutrophil gelatinase‐associated lipocalin. MMP‐9, matrix metalloproteinases‐9. IS, ischemic stroke

In this part, we determined plasma CEACAM1 levels among IS groups and the control group. The level of plasma CEACAM1 in the subacute group showed a significant increase compared with that in the healthy control group (*p* = 0.016, Figure 3A). No statistical differences were noticed between subacute group and the other groups (*p* > 0.05). Moreover, the expression of CEACAM1 on the surface of neutrophils in the subacute group was closely correlated with the plasma CEACAM1 content (*p* = 0.001, *r* = 0.614, Figure 3B).

### 3.4 Determination of poor‐plate plasma MMP‐9 and NGAL

We determined the plasma MMP‐9 concentration in different groups. Significant increase was noticed in the plasma MMP‐9 concentration in the subacute group when comparing with that of the control group (*p* = 0.005) and acute group (*p* = 0.020, Figure 3C). There were no statistical differences among the other groups (*p* > 0.05).

We also determined the level of plasma NAGL in different groups. Compared with the healthy control individuals, a significant elevation was noticed in the plasma NAGL in the subacute group (*p* = 0.001) and stable group (*p* < 0.001, Figure 3D). There were no statistical differences in the plasma NAGL between the other groups (*p* > 0.05).

### 3.5 Determination of plasma soluble TNF and IL‐10

**FIGURE 4:**
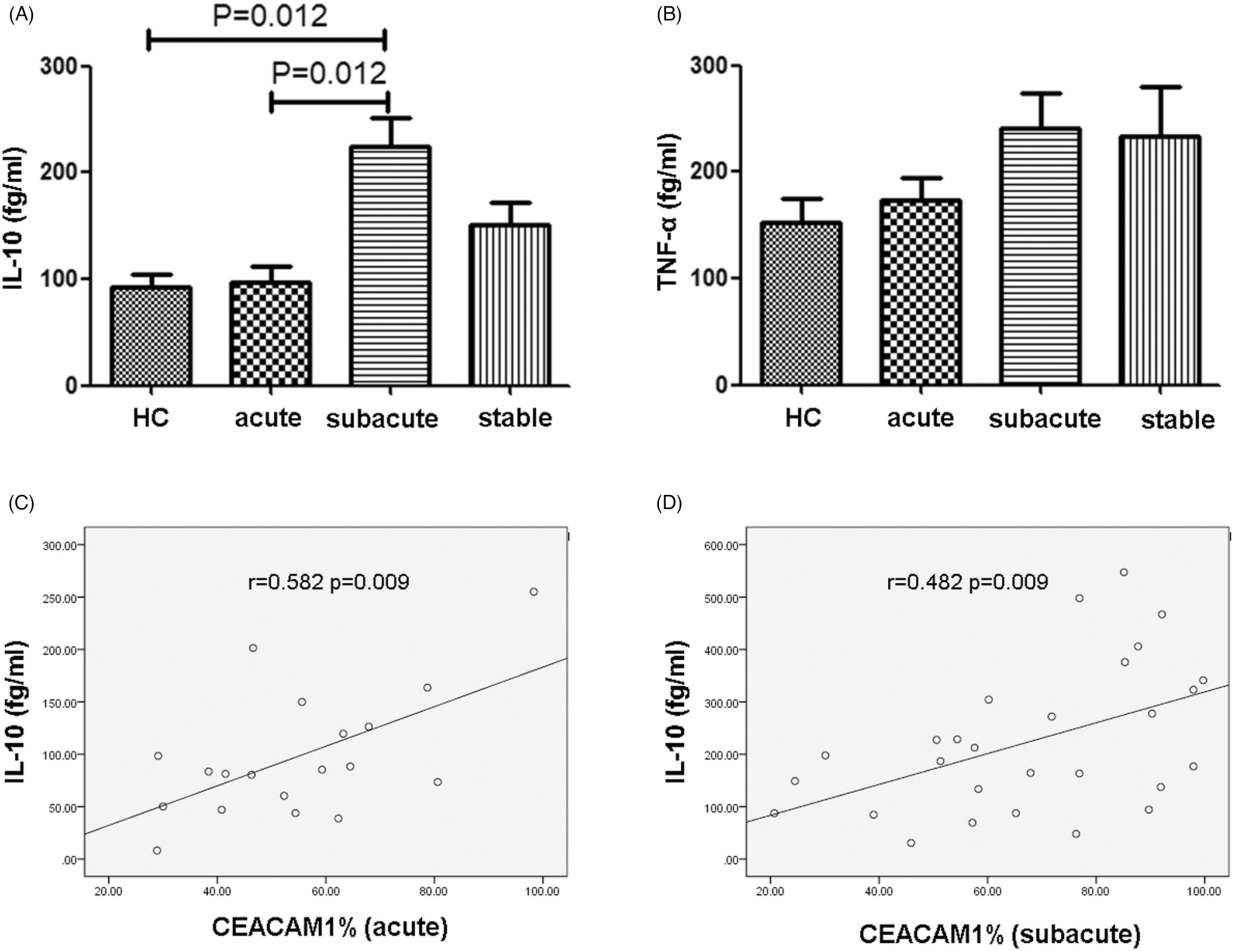
Content of IL‐10 and TNF in the plasma of patients with IS. (A) Comparison of plasma IL‐10 concentration in the IS patients and healthy control. (B) Comparison of plasma TNF concentration in the IS patients and healthy control. Statistical analysis was performed by the Mann–Whitney test. (C) Correlation analysis between plasma IL‐10 content and CEACAM1 positive neutrophils in the acute group. (D) Correlation analysis between plasma IL‐10 content and CEACAM1 positive neutrophils in the subacute group. Correlation determined by the Pearson correlation analysis. IL‐10, interleukin 10. TNF, tumor necrosis factor. IS, ischemic stroke

We also determined the level of plasma TNF and IL‐10 in different groups. A significant increase was noticed in the plasma IL‐10 concentration in the subacute group when comparing with that of the control group (*p* = 0.012) and acute group (*p* = 0.012, Figure 4A). There were no statistical differences among the other groups (*p* > 0.05). Moreover, the expression of CEACAM1 on the surface of neutrophils in the acute and subacute group was closely correlated with the plasma IL‐10 content (*p* = 0.009, *r* = 0.582 and *p* = 0.009, *r* = 0.482, respectively，Figure 4C and D). There were no statistical differences in the concentration of TNF among the different groups (*p* > 0.05, Figure 4B), and TNF contents in all the groups were not correlated with levels of CEACAM1 expression in neutrophils.

### 3.6 MMP‐9 production by CEACAM1‐positive and negative neutrophils

To confirm the correlation between expression of CEACAM1 and the production of MMP‐9 of neutrophils, the neutrophils were subjected to lipopolysaccharide (LPS) stimulation among the patients from the subacute group. Flow cytometry was utilized to determine the MFI of MMP‐9 in the CEACAM1‐positive and negative neutrophils. As shown in Figure S2, MMP‐9 was expressed in both CEACAM1‐positive and negative neutrophils. However, there were no statistical differences in the MFI of the two subtypes of neutrophils (*p* > 0.05).

## 4 DISCUSSION

Cerebral edema after BBB breakdown is considered an important cause for the severe morbidity and mortality after IS, serving as an important cause for the early stroke‐related death.^36^ Besides, SAI is the major cause for the late‐stage mortality in IS.^37^ Increasing evidence suggested that functional disorder of the immunocytes was an important cause for the early‐stage BBB injury and subsequent SAI among IS patients.^12^ However, the roles of neutrophils in the pathogenesis of IS have not been well defined. According to previous studies, MMP‐9 was implicated in the BBB breakdown and formation of vasogenic edema after IS.^38^, ^39^ In an IS mice model, CEACAM1 was reported to involve in controlling the secretion of MMP‐9 by neutrophils in post‐ischemic inflammation in the BBB after IS.^22^ This demonstrated that CEACAM1 may inhibit the neutrophil‐mediated tissue damages and breakdown of BBB in focal cerebral ischemia. In this study, we determined the expression of CEACAM1 in leukocytes of IS patients. Our data confirmed that neutrophils were the major source of CEACAM1 in peripheral blood. CEACAM1 expression on the surface of peripheral neutrophils of IS patients at each stage was significantly higher than that of the healthy control. Then, we measured the expression of plasma CEACAM1, MMP‐9, and NGAL content, which showed that the plasma MMP‐9 level in patients at subacute phases was significantly higher than that of the acute stage and the healthy control. Interestingly, there were no statistical differences in the plasma MMP‐9 between the healthy control and the acute group. This implied that there was persistent elevation of MMP‐9 within several days after onset of IS, which may be closely related to the subsequent brain edema and nervous injuries. Ludewig Peter and his colleges described a significant increase in the expression of MMP‐9 in CEACAM1^−/−^ IS mice model compared with that of the wild‐type mice and the results were validated with stimulation of neutrophils under in vitro conditions showing increased MMP‐9 secretion in CEACAM1‐deficient neutrophils 120 min afterward.^22^ Our data suggested a significant decline of cell viability in CEACAM1‐positive neutrophils compared with CEACAM1‐negative cells. Nevertheless, in this study, upon LPS stimulation, no statistical differences were noticed in the MMP‐9 expression in CEACAM1‐positive and negative neutrophils in IS patients. Neutrophils are main resource of MMP‐9 after IS onset.^40^ On this basis, we speculated that CEACAM1 may regulated neutrophil cell viability and finally influenced MMP‐9 secretion.

Previous study indicated that IS patients with increased plasma NGAL showed poorer prognosis.^41^ Our data on the IS patients were in line with the study by Hochmeister Sonja et al,^41^ in which a significant increase was noticed in the plasma NGAL at the subacute stage. Evidences indicated that NGAL, MMP‐9/NGAL, and MMP‐9 were co‐expressed in the activated monocytes and neutrophils. Esidue in the PEX domain of MMP‐9.^32^ NGAL could regulate the activity of MMP‐9. In a previous study, the Cys‐87 in NGAL could form a disulphide bond with an as yet unidentified cysteine residue in the PEX domain of MMP‐9.^32^ In the presence of NGAL, the degradation of MMP‐9 was obviously inhibited, which led to the preserve of the MMP‐9 activity.^33^ In this study, despite the fact that MMP‐9 elevation was merely noticed at the subacute stage, there was persistent increase in plasma NGAL. We speculated from our data that high concentration of NGAL may activate or preserve the MMP‐9 activity. This may contribute to the fact that the activity level of MMP‐9 was higher than the normal level even its concentration was not higher in the stable period. On this basis, we speculated that NGAL and MMP‐9 played important roles in BBB injury.

In this study, we examined plasma TNF‐α and IL‐10, our study showed that expression evaluation of cytokines IL‐10 and neutrophil CEACAM1 expression was positively correlated with plasma IL‐10 level in patients at IS acute and subacute phases. The positive relation between CEACAM1 and IL‐10 may suggest that CEACAM1 played roles in regulation of downstream cytokine secretion.^42^ Previous in vitro and in vivo models of IS showed IL‐10‐mediated neuroprotection directly and indirectly,^43^ but the specific mechanism remained unclear. Related studies are needed to further illustrate the exact mechanism in the future.

In a previous study, CEACAM1 was considered to be related to the proliferation of neutrophils and granulopoiesis^26^ as well as the secretion of cytokines and apoptosis.^25^, ^28^ In this study, the level of CEACAM1 on the surface of neutrophils in IS patients was significantly higher compared with the healthy control. Meanwhile we also discovered that there was higher MFI of apoptosis markers in CEACAM1‐positive neutrophils compared with negative counterparts. On this basis, we speculated that CEACAM1 might involve in regulation of CEACAM1‐induced neutrophil proliferation and granulopoiesis.

CEACAM1 on the surface of neutrophils in the peripheral blood is an important source of plasma CEACAM1.^44^ Our data indicated that plasma CEACAM1 was positively correlated with the CEACAM1 on the neutrophils in the peripheral blood of subacute stage IS patients. To our best knowledge, TIM‐3 serving as an important immune checkpoint factor of T lymphocytes is mainly responsible for the negative regulation of T lymphocytes.^45^ As a ligand of TIM‐3, CEACAM1 could mediate the function of TIM‐3 modulated T lymphocytes.^46^, ^47^ Thus, it could affect the TIM‐3 positive T lymphocyte proliferation,^27^ function^48^ and secretion of cytokines.^29^ Our previous study indicated that there was increased TIM‐3 on the peripheral blood T lymphocytes in the IS.^30^ We speculated that CEACAM1 could bind with TIM‐3 through the T lymphocyte surface, which may induce injury of immune function of lymphocytes at the late‐stage IS patients and finally result in increased risk of infection. In the future, more studies are required to validate this speculation.

MMP‐9 deficiency has been proved to increase in vitro apoptosis in multiple tissues.^49^ MMP‐9 induced increase in expression of FasL in diabetes wound repair.^50^ In kidney development, MMP‐9 was discovered to protect mesenchymal cells from apoptosis.^49^, ^51^ The process may involve various downstream molecules of MMP‐9. In a study on post‐ischemic cerebral injury, evidence showed that reduction in expression of MMP‐9 may lead to protective effects on BBB injury and neuronal apoptosis of adipocyte fatty acid‐binding protein (A‐FABP) deficiency.^52^ MMP‐9 may also play the role of apoptosis inhibition through hedghog pathway.^53^ There has been evidence about CEACAM1‐mediated apoptosis in exacerbates hypoxic cardiomyocyte injury and post‐infarction cardiac remodeling,^54^ and early tumor development.^55^ In this study, we found significant reduction in cell viability in CEACAM1‐positive neutrophils compared with CEACAM1‐negative ones, and we also noticed elevation of MMP‐9 at the subacute stage. It is speculated that after onset of cerebral ischemic stroke, increased CEACAM1 level induced more apoptosis of neutrophils. MMP‐9 elevation may due to negative feed back mechanism in response to elevated apoptosis level. However, we lack evidence to support such speculation.

CEACAM1 has been proved to be implicated in vascular physiology and pathophysiology, which includes vessel formation, endothelial barrier function regulation, vascular remodeling, and stabilization.^56^ In CEACAM1^−/−^ mice, multiple pathways involved in NADPH oxidases elevation and activation.^57^ Increased signaling by VEGFR‐2 and TNF‐α/NF‐κB pathway and the renin‐angiotensin system activation was both observed in CEACAM1^−/−^ mice, suggesting that CEACAM1 played a significant role in endothelial oxidation.^56^ CEACAM1, expressed on both human and mice platelets, was also found to inhibit platelet activation. In previous study, CEACAM1^−/−^ mice were observed to be more susceptible to thrombosis, and CEACAM1^−/−^ platelets displayed enhanced type I collagen and CRP‐mediated platelet aggregation, adhesion, and dense granule secretion, which suggested that CEACAM1 could negatively regulate platelet‐collagen interactions and thrombus growth in vitro and in vivo.^58^

In summary, CEACAM1 expression level in neutrophils was higher in IS patients of different stages than that in normal individuals. Additionally, significant elevation was noticed in the plasma CEACAM1, MMP‐9, and NGAL in IS patients. CEACAM1 may serve as an important inhibitory regulator for mediating the early‐stage BBB injury induced by MMP‐9. Nevertheless, persistent high expression of plasma NGAL would combine with MMP‐9, which may contribute to the persistent injury of BBB. Moreover, as a ligand of TIM‐3, CEACAM1 may involve in the negative immunoregulation of T lymphocytes, which may lead to severe immune dysfunction and subsequent infections. In the future, attempts should be made to verify the interaction among CEACAM1, MMP‐9, and NGAL, and to further illustrate the roles of CEACAM1 in the IS, ischemia–reperfusion injury and secondary infection in the patients and animal models.

However, there are some limitations in this study. The study failed to gather blood samples at different stages from each patient. Though change in CEACAM1 and related markers has been classified, the pathophysiological significance of CEACAM1 elevation has not been confirmed. Besides, the small sample size in the study may interfere in the results and needs larger sample size to verify the discoveries.

In this study, rapid elevation of neutrophil CEACAM1 and change in levels of associated inflammatory mediators in the plasma were observed at the acute stage and subacute stage of cerebral IS. The results implied that CEACAM1 may serve as an inhibitory regulator affecting the progression of focal cerebral ischemia, especially involved in the BBB injury progress.

## AUTHOR CONTRIBUTIONS

YZ carried out the flow cytometric analysis, participated in the design of the study, and helped in drafting the article. YW determined the relations among CEACAM1, NGAL, and MMP‐9, participated in the design of the study and revised the article. WW carried out supplemental experiments and revised and polished the article. PL and SS participated in the design of the study, and helped in drafting the article. MH analyzed and interpreted the results, and revised the article. YY participated in the sample collection. QX and ZC conceived the study, participated in its design and coordination, and helped in drafting the article. All authors read and approved the final article. Yi Zhang, Yijie Wang, and Wei Wu contributed equally to this work.

## CONFLICT OF INTEREST

The authors report no conflicts of interest in this work.

## PATIENT CONSENT STATEMENT

Written informed consent was obtained from all the participants.

## Supporting information


Figure S1

Figure S2

Appendix S1

## Data Availability

The data that support the findings of this study are available from the corresponding author upon reasonable request.

## References

[1] Feigin VL , Nguyen G , Cercy K , et al. Global, Regional, and Country‐specific lifetime risks of stroke, 1990 and 2016. N Engl J Med. 2018;379(25):2429‐2437. 30575491 10.1056/NEJMoa1804492 PMC6247346

[2] Global, regional, and national disability‐adjusted life‐years (DALYs) for 359 diseases and injuries and healthy life expectancy (HALE) for 195 countries and territories, 1990–2017: a systematic analysis for the global burden of disease study 2017. Lancet. 2018;392(10159):1859‐1922. 30415748 10.1016/S0140-6736(18)32335-3 PMC6252083

[3] Pandian JD , Gall SL , Kate MP , et al. Prevention of stroke: a global perspective. Lancet. 2018;392(10154):1269‐1278. 30319114 10.1016/S0140-6736(18)31269-8

[4] Macrez R , Ali C , Toutirais O , et al. Stroke and the immune system: from pathophysiology to new therapeutic strategies. Lancet Neurol. 2011;10(5):471‐480. 21511199 10.1016/S1474-4422(11)70066-7

[5] Iadecola C , Anrather J . The immunology of stroke: from mechanisms to translation. Nat Med. 2011;17(7):796‐808. 21738161 10.1038/nm.2399 PMC3137275

[6] Chamorro A , Urra X , Planas AM . Infection after acute ischemic stroke: a manifestation of brain‐induced immunodepression. Stroke. 2007;38(3):1097‐1103. 17255542 10.1161/01.STR.0000258346.68966.9d

[7] Dirnagl U , Klehmet J , Braun JS , et al. Stroke‐induced immunodepression: experimental evidence and clinical relevance. Stroke. 2007;38(2 Suppl):770‐773. 17261736 10.1161/01.STR.0000251441.89665.bc

[8] Famakin BM . The immune response to acute focal cerebral ischemia and associated post‐stroke immunodepression: a focused review. Aging Dis. 2014;5(5):307‐326. 25276490 10.14336/AD.2014.0500307 PMC4173797

[9] Hoffmann S , Harms H , Ulm L , et al. Stroke‐induced immunodepression and dysphagia independently predict stroke‐associated pneumonia ‐ The PREDICT study. J Cereb Blood Flow Metab. 2017;37(12):3671‐3682. 27733675 10.1177/0271678X16671964 PMC5718319

[10] Liu DD , Chu SF , Chen C , Yang PF , Chen NH , He X . Research progress in stroke‐induced immunodepression syndrome (SIDS) and stroke‐associated pneumonia (SAP). Neurochem Int. 2018;114:42‐54. 29317279 10.1016/j.neuint.2018.01.002

[11] Ingeman A , Andersen G , Hundborg HH , Svendsen ML , Johnsen SP . In‐hospital medical complications, length of stay, and mortality among stroke unit patients. Stroke. 2011;42(11):3214‐3218. 21868737 10.1161/STROKEAHA.110.610881

[12] Amantea D , Micieli G , Tassorelli C , et al. Rational modulation of the innate immune system for neuroprotection in ischemic stroke. Front Neurosci. 2015;9:147. 25972779 10.3389/fnins.2015.00147 PMC4413676

[13] Gelderblom M , Leypoldt F , Steinbach K , et al. Temporal and spatial dynamics of cerebral immune cell accumulation in stroke. Stroke. 2009;40(5):1849‐1857. 19265055 10.1161/STROKEAHA.108.534503

[14] Jin R , Yang G , Li G . Inflammatory mechanisms in ischemic stroke: role of inflammatory cells. J Leukoc Biol. 2010;87(5):779‐789. 20130219 10.1189/jlb.1109766 PMC2858674

[15] Rosenberg GA , Estrada EY , Dencoff JE . Matrix metalloproteinases and TIMPs are associated with blood‐brain barrier opening after reperfusion in rat brain. Stroke. 1998;29(10):2189‐2195. 9756602 10.1161/01.str.29.10.2189

[16] Terao S , Yilmaz G , Stokes KY , Ishikawa M , Kawase T , Granger DN . Inflammatory and injury responses to ischemic stroke in obese mice. Stroke. 2008;39(3):943‐950. 18239178 10.1161/STROKEAHA.107.494542

[17] Rodrigues SF , Granger DN . Blood cells and endothelial barrier function. Tissue Barriers. 2015;3(1–2):e978720. 25838983 10.4161/21688370.2014.978720 PMC4372023

[18] Ning M , Furie KL , Koroshetz WJ , et al. Association between tPA therapy and raised early matrix metalloproteinase‐9 in acute stroke. Neurology. 2006;66(10):1550‐1555. 16717217 10.1212/01.wnl.0000216133.98416.b4

[19] Copin JC , Goodyear MC , Gidday JM , et al. Role of matrix metalloproteinases in apoptosis after transient focal cerebral ischemia in rats and mice. Eur J Neurosci. 2005;22(7):1597‐1608. 16197500 10.1111/j.1460-9568.2005.04367.x

[20] Rong T , He M , Hua Y , Chen D , Chen M . Associations of interleukin 10, matrix metallopeptidase 9, and legumain with blood pressure variability and neurologic outcomes in patients with ischemic stroke. Int J Gen Med. 2020;13:1595‐1602. 33364822 10.2147/IJGM.S285003 PMC7751781

[21] Tang Y , Xu H , Du X , et al. Gene expression in blood changes rapidly in neutrophils and monocytes after ischemic stroke in humans: a microarray study. J Cereb Blood Flow Metab. 2006;26(8):1089‐1102. 16395289 10.1038/sj.jcbfm.9600264

[22] Ludewig P , Sedlacik J , Gelderblom M , et al. Carcinoembryonic antigen‐related cell adhesion molecule 1 inhibits MMP‐9‐mediated blood‐brain‐barrier breakdown in a mouse model for ischemic stroke. Circ Res. 2013;113(8):1013‐1022. 23780386 10.1161/CIRCRESAHA.113.301207

[23] Gray‐Owen SD , Blumberg RS . CEACAM1: contact‐dependent control of immunity. Nat Rev Immunol. 2006;6(6):433‐446. 16724098 10.1038/nri1864

[24] Chen T , Zimmermann W , Parker J , Chen I , Maeda A , Bolland S . Biliary glycoprotein (BGPa, CD66a, CEACAM1) mediates inhibitory signals. J Leukoc Biol. 2001;70(2):335‐340. 11493628

[25] Singer BB , Klaile E , Scheffrahn I , et al. CEACAM1 (CD66a) mediates delay of spontaneous and Fas ligand‐induced apoptosis in granulocytes. Eur J Immunol. 2005;35(6):1949‐1959. 15909305 10.1002/eji.200425691

[26] Pan H , Shively JE . Carcinoembryonic antigen‐related cell adhesion molecule‐1 regulates granulopoiesis by inhibition of granulocyte colony‐stimulating factor receptor. Immunity. 2010;33(4):620‐631. 21029969 10.1016/j.immuni.2010.10.009 PMC3765078

[27] Chen CJ , Shively JE . The cell‐cell adhesion molecule carcinoembryonic antigen‐related cellular adhesion molecule 1 inhibits IL‐2 production and proliferation in human T cells by association with Src homology protein‐1 and down‐regulates IL‐2 receptor. J Immunol. 2004;172(6):3544‐3552. 15004155 10.4049/jimmunol.172.6.3544

[28] Lu R , Pan H , Shively JE . CEACAM1 negatively regulates IL‐1β production in LPS activated neutrophils by recruiting SHP‐1 to a SYK‐TLR4‐CEACAM1 complex. PLoS Pathog. 2012;8(4):e1002597. 22496641 10.1371/journal.ppat.1002597 PMC3320586

[29] Nagaishi T , Iijima H , Nakajima A , et al. Role of CEACAM1 as a regulator of T cells. Ann N Y Acad Sci. 2006;1072:155‐175. 17057197 10.1196/annals.1326.004

[30] Zhang Y , Wei L , Du Y , et al. Association between programed cell death‐1 and CD4(+) T cell alterations in different phases of ischemic stroke patients. Front Cell Neurosci. 2018;12:170. 30013463 10.3389/fncel.2018.00170 PMC6036251

[31] Das M , Zhu C , Kuchroo VK . Tim‐3 and its role in regulating anti‐tumor immunity. Immunol Rev. 2017;276(1):97‐111. 28258697 10.1111/imr.12520 PMC5512889

[32] Chakraborty S , Kaur S , Guha S , Batra SK . The multifaceted roles of neutrophil gelatinase associated lipocalin (NGAL) in inflammation and cancer. Biochim Biophys Acta. 2012;1826(1):129‐169. 22513004 10.1016/j.bbcan.2012.03.008 PMC3362670

[33] Bouchet S , Bauvois B . Neutrophil gelatinase‐associated lipocalin (NGAL), pro‐matrix metalloproteinase‐9 (pro‐MMP‐9) and their complex Pro‐MMP‐9/NGAL in leukaemias. Cancers (Basel). 2014;6(2):796‐812. 24713998 10.3390/cancers6020796 PMC4074804

[34] Asplund K , Bonita R , Kuulasmaa K , et al. Multinational comparisons of stroke epidemiology. Evaluation of case ascertainment in the WHO MONICA stroke study. world health organization monitoring trends and determinants in cardiovascular disease. Stroke. 1995;26(3):355‐360. 7886706 10.1161/01.str.26.3.355

[35] A systems approach to immediate evaluation and management of hyperacute stroke. experience at eight centers and implications for community practice and patient care. the national institute of neurological disorders and stroke (NINDS) rt‐PA stroke study group. Stroke. 1997;28(8):1530‐1540. 9259745 10.1161/01.str.28.8.1530

[36] Vahedi K , Hofmeijer J , Juettler E , et al. Early decompressive surgery in malignant infarction of the middle cerebral artery: a pooled analysis of three randomised controlled trials. Lancet Neurol. 2007;6(3):215‐222. 17303527 10.1016/S1474-4422(07)70036-4

[37] Suda S , Aoki J , Shimoyama T , et al. Stroke‐associated infection independently predicts 3‐month poor functional outcome and mortality. J Neurol. 2018;265(2):370‐375. 29249057 10.1007/s00415-017-8714-6

[38] Rosenberg GA , Yang Y . Vasogenic edema due to tight junction disruption by matrix metalloproteinases in cerebral ischemia. Neurosurg Focus. 2007;22(5):E4. 10.3171/foc.2007.22.5.5 17613235

[39] Gasche Y , Fujimura M , Morita‐Fujimura Y , et al. Early appearance of activated matrix metalloproteinase‐9 after focal cerebral ischemia in mice: a possible role in blood‐brain barrier dysfunction. J Cereb Blood Flow Metab. 1999;19(9):1020‐1028. 10478654 10.1097/00004647-199909000-00010

[40] Vandooren J , Van den Steen PE , Opdenakker G . Biochemistry and molecular biology of gelatinase B or matrix metalloproteinase‐9 (MMP‐9): the next decade. Crit Rev Biochem Mol Biol. 2013;48(3):222‐272. 23547785 10.3109/10409238.2013.770819

[41] Hochmeister S , Engel O , Adzemovic MZ , et al. Lipocalin‐2 as an infection‐related biomarker to predict clinical outcome in ischemic stroke. PLoS One. 2016;11(5):e0154797. 27152948 10.1371/journal.pone.0154797 PMC4859492

[42] Kany S , Vollrath JT , Relja B . Cytokines in inflammatory disease. Int J Mol Sci. 2019;20(23):6008. 10.3390/ijms20236008 PMC6929211 31795299

[43] Garcia JM , Stillings SA , Leclerc JL , et al. Role of Interleukin‐10 in Acute Brain Injuries. Front Neurol. 2017;8:244. 28659854 10.3389/fneur.2017.00244 PMC5466968

[44] Kuroki M , Matsuo Y , Kinugasa T , Matsuoka Y . Augmented expression and release of nonspecific cross‐reacting antigens (NCAs), members of the CEA family, by human neutrophils during cell activation. J Leukoc Biol. 1992;52(5):551‐557. 1431566 10.1002/jlb.52.5.551

[45] Tang R , Rangachari M , Kuchroo VK . Tim‐3: A co‐receptor with diverse roles in T cell exhaustion and tolerance. Semin Immunol. 2019;42:101302. 31604535 10.1016/j.smim.2019.101302

[46] Huang YH , Zhu C , Kondo Y , et al. CEACAM1 regulates TIM‐3‐mediated tolerance and exhaustion. Nature. 2015;517(7534):386‐390. 25363763 10.1038/nature13848 PMC4297519

[47] Sabatos‐Peyton CA , Nevin J , Brock A , et al. Blockade of Tim‐3 binding to phosphatidylserine and CEACAM1 is a shared feature of anti‐Tim‐3 antibodies that have functional efficacy. Onco Targets Ther. 2018;7(2):e1385690. 10.1080/2162402X.2017.1385690 PMC5749620 29308307

[48] Nakajima A , Iijima H , Neurath MF , et al. Activation‐induced expression of carcinoembryonic antigen‐cell adhesion molecule 1 regulates mouse T lymphocyte function. J Immunol. 2002;168(3):1028‐1035. 11801635 10.4049/jimmunol.168.3.1028

[49] Arnould C , Lelievre‐Pegorier M , Ronco P , et al. MMP9 limits apoptosis and stimulates branching morphogenesis during kidney development. J Am Soc Nephrol. 2009;20(10):2171‐2180. 19713309 10.1681/ASN.2009030312 PMC2754105

[50] Liang Y , Yang C , Lin Y , et al. Matrix metalloproteinase 9 induces keratinocyte apoptosis through FasL/Fas pathway in diabetic wound. Apoptosis. 2019;24(7–8):542‐551. 30949883 10.1007/s10495-019-01536-w

[51] Bengatta S , Arnould C , Letavernier E , et al. MMP9 and SCF protect from apoptosis in acute kidney injury. J Am Soc Nephrol. 2009;20(4):787‐797. 19329763 10.1681/ASN.2008050515 PMC2663840

[52] Liao B , Geng L , Zhang F , et al. Adipocyte fatty acid‐binding protein exacerbates cerebral ischaemia injury by disrupting the blood‐brain barrier. Eur Heart J. 2020;41(33):3169‐3180. 32350521 10.1093/eurheartj/ehaa207 PMC7556749

[53] Wu MY , Gao F , Yang XM , et al. Matrix metalloproteinase‐9 regulates the blood brain barrier via the hedgehog pathway in a rat model of traumatic brain injury. Brain Res. 2020;1727:146553. 31734396 10.1016/j.brainres.2019.146553

[54] Wang Y , Chen Y , Yan Y , et al. Loss of CEACAM1, a tumor‐associated factor, attenuates post‐infarction cardiac remodeling by inhibiting apoptosis. Sci Rep. 2016;6:21972. 26911181 10.1038/srep21972 PMC4766464

[55] Nittka S , Bohm C , Zentgraf H , et al. The CEACAM1‐mediated apoptosis pathway is activated by CEA and triggers dual cleavage of CEACAM1. Oncogene. 2008;27(26):3721‐3728. 18278069 10.1038/sj.onc.1211033

[56] Rueckschloss U , Kuerten S , Ergün S . The role of CEA‐related cell adhesion molecule‐1 (CEACAM1) in vascular homeostasis. Histochem Cell Biol. 2016;146(6):657‐671. 27695943 10.1007/s00418-016-1505-9

[57] Najjar SM , Ledford KJ , Abdallah SL , et al. Ceacam1 deletion causes vascular alterations in large vessels. Am J Physiol Endocrinol Metab. 2013;305(4):E519‐E529. 23800882 10.1152/ajpendo.00266.2013 PMC3891225

[58] Wong C , Liu Y , Yip J , et al. CEACAM1 negatively regulates platelet‐collagen interactions and thrombus growth in vitro and in vivo. Blood. 2009;113(8):1818‐1828. 19008452 10.1182/blood-2008-06-165043

