# Supplementary figures and images for: Elevation of neutrophil carcinoembryonic antigen‐related cell adhesion molecule 1 associated with multiple inflammatory mediators was related to different clinical stages in ischemic stroke patients
Source: J Clin Lab Anal. 2022 Jun 3;36(7):e24526. doi: 10.1002/jcla.24526 (PMC9279952; doi:10.1002/jcla.24526)

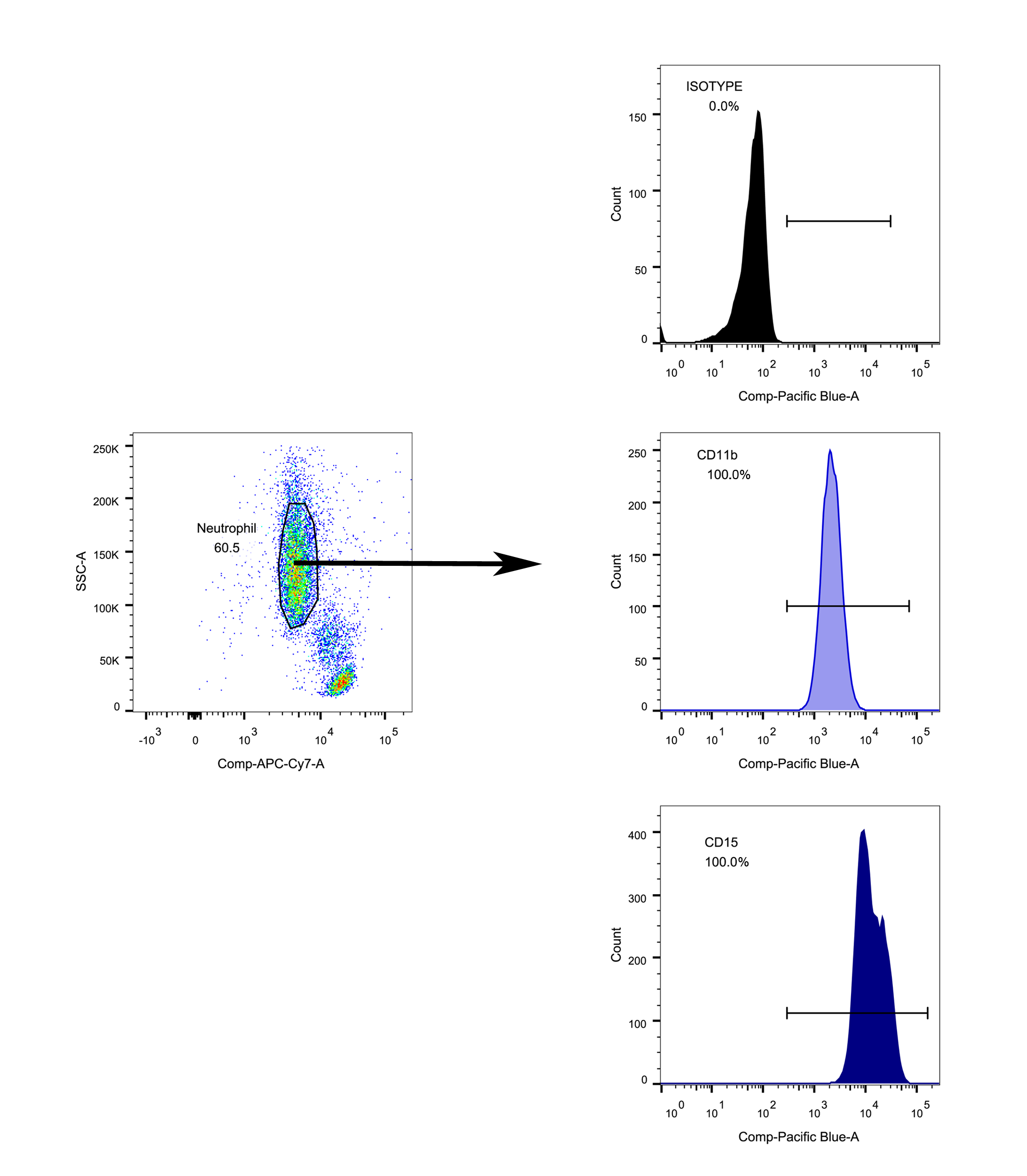

Supplement: Supplementary file 1 — Figure S1 [file JCLA-36-e24526-s003.TIF]

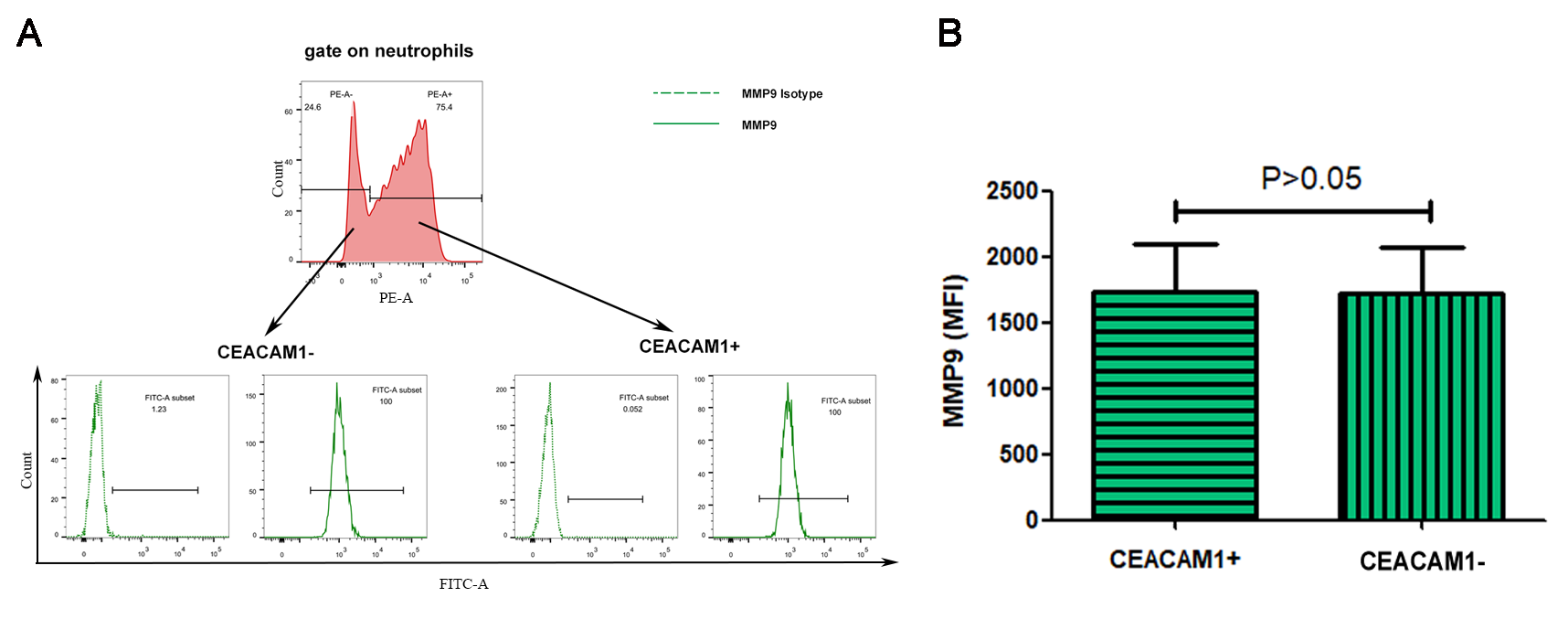

Supplement: Supplementary file 2 — Figure S2 [file JCLA-36-e24526-s001.tif]
