# Supplementary material for: Elevation of neutrophil carcinoembryonic antigen‐related cell adhesion molecule 1 associated with multiple inflammatory mediators was related to different clinical stages in ischemic stroke patients
Source: J Clin Lab Anal. 2022 Jun 3;36(7):e24526. doi: 10.1002/jcla.24526 (PMC9279952; doi:10.1002/jcla.24526)
Supplement: Supplementary file 3 — Appendix S1 [file JCLA-36-e24526-s002.docx]

Figure S1 The neutrophils were gated by CD45 and SSC, and verified by CD11b and CD15. SSC, side scatter.

Figure S2 Expression of MMP-9 in CEACAM1 positive and negative neutrophils in patients at subacute stage after treating with LPS (100 ng/mL) for 2 h. (A) Gating strategy. The dotted line represented the sample isotype, and the solid line represented the measurement value. (B). MFI showed no statistical differences in the MMP-9 in the CEACAM1 positive and negative neutrophils (n = 5). Statistical analysis was performed by the Mann–Whitney test. MMP-9, matrix metalloproteinases-9. LPS, lipopolysaccharide. MFI, Mean fluorescence intensity. CEACAM1, carcinoembryonic antigen-related cell adhesion molecule 1.
